# Organizational innovation climate and nurses’ innovation behavior in a specialized oncology hospital: the chain mediation of achievement motivation and creative self-efficacy

**DOI:** 10.1186/s12912-025-03595-8

**Published:** 2025-07-21

**Authors:** Xue Fu, Le Xia, Liping Chen, Mei Zhong, Yang Liu, Wenhao He, Baojia Luo, Linmin Chen, Yuying Fan, Huiying Qin

**Affiliations:** 1 Department of Nursing, State Key Laboratory of Oncology in South China, Guangdong Provincial Clinical Research Center for Cancer, Sun Yat-sen University Cancer Center, Guangzhou, 510060 PR China; 2 Department of Outpatient, State Key Laboratory of Oncology in South China, Guangdong Provincial Clinical Research Center for Cancer, Sun Yat- sen University Cancer Center, Guangzhou, 510060 PR China; 3 Department of Radiation Oncology, State Key Laboratory of Oncology in South China, Guangdong Provincial Clinical Research Center for Cancer, Sun Yat-sen University Cancer Center, Guangzhou, 510060 PR China; 4 Department of Critical Care Medicine, State Key Laboratory of Oncology in South China, Guangdong Provincial Clinical Research Center for Cancer, Sun Yat-sen University Cancer Center, Guangzhou, 510060 PR China; 5 Department of Anesthesiology, State Key Laboratory of Oncology in South China, Guangdong Provincial Clinical Research Center for Cancer, Sun Yat- sen University Cancer Center, Guangzhou, 510060 PR China; 6 Department of Bone and Soft Tissue, State Key Laboratory of Oncology in South China, Guangdong Provincial Clinical Research Center for Cancer, Sun Yat-sen University Cancer Center, Guangzhou, 510060 PR China; 7 Department of Colorectal Surgery, State Key Laboratory of Oncology in South China, Guangdong Provincial Clinical Research Center for Cancer, Sun Yat-sen University Cancer Center, Guangzhou, 510060 PR China; 8 Department of Nasopharyngeal Carcinoma, State Key Laboratory of Oncology in South China, Guangdong Provincial Clinical Research Center for Cancer, Sun Yat-sen University Cancer Center, Guangzhou, 510060 PR China

**Keywords:** Achievement motivation, Creative self-efficacy, Organizational innovation climate, Nurses’ innovation behavior, Mediating effect

## Abstract

**Objective:**

This study explains the impact of organizational innovation climate on nurses’ innovation behavior in a tertiary specialized oncology hospital, while examining the chain mediation roles of achievement motivation and creative self-efficacy.

**Methods:**

In this online cross-sectional survey, 857 nurses from a tertiary specialized oncology hospital in Guangdong province, China were selected by convenience sampling method. SPSS Statistics v26.0 and AMOS v29.0 were used for data analysis. The reporting followed the STROBE checklist.

**Results:**

The nurses’ innovation behavior was below the medium level. Organizational innovation climate was significantly positively related to nurses’ innovation behavior, achievement motivation, and creative self-efficacy. Achievement motivation was positively correlated with both creative self-efficacy and nurses’ innovation behavior. Creative self-efficacy was positively correlated with nurses’ innovation behavior. Mediation analysis identified two pathways: creative self-efficacy independently mediated the relationship between organizational innovation climate and nurses’ innovation behavior, while achievement motivation and creative self-efficacy served as a chain mediator between the organizational innovation climate and nurses’ innovation behavior.

**Conclusion:**

Our study highlights the critical role of organizational innovation climate in enhancing nurses’ innovation behavior in specialized oncology hospitals. Specifically, it demonstrates that achievement motivation and creative self-efficacy play a chain mediation role between organizational innovation climate and nurses’ innovation behavior. Healthcare administrators should prioritize constructing innovation-supportive climates, implement achievement motivation interventions, and strengthen creative self-efficacy development programs to systematically foster nurses’ innovation capabilities in oncology settings.

**Supplementary Information:**

The online version contains supplementary material available at 10.1186/s12912-025-03595-8.

## Background

Amidst accelerated technological advancement and escalating public health demands, the healthcare industry confronts unprecedented challenges in service delivery and quality expectations. In China, the “Health China 2023” strategic framework positions healthcare system reform and technological innovation as critical drivers for enhancing service capacity and addressing population health requirements [1]. This recognition establishes innovation as an essential competency for maintaining operational effectiveness within intricate clinical environments. Nursing professionals, given their frontline patient engagement and sustained care provision roles, possess unique vantage points to detect systemic gaps that frequently serve as catalysts for innovation [2, 3]. Nurses’ innovation behavior is operationally defined as a systematic process involving identifying and developing new methods, techniques, or work approaches, and implementing them into practices after gaining the support from others [4]. Its ultimate target is to promote health, prevent diseases, and optimize care quality [4]. The International Council of Nurses (ICN) further substantiates this priority by identifying nursing innovation as an indispensable evolutionary force within modern healthcare ecosystems [5].

According to global epidemiological data from the World Health Organization, there were about 20 million new cancer cases diagnosed in 2022, with projections suggesting a surge to 35 million cases by 2050 [6]. In China, there were 4.82 million new cancer cases [6], establishing oncological disease management as a formidable global health challenge. Specialized oncology nursing expertise constitutes a cornerstone in delivering optimal oncological care [7]. Nevertheless, nursing teams in specialized oncology hospitals confront multidimensional clinical demands and occupational stressors, manifested through elevated burnout prevalence that jeopardizes care standards and workforce stability [8–10]. Empirical evidence substantiates that nurses’ innovation behavior generates multidimensional benefits, including optimized clinical workflows, accelerated care delivery timelines, enhanced resource allocation efficiency, and controlled healthcare expenditures [11, 12]. Furthermore, it is of great significance in enhancing occupational self-concept, achieving career fulfillment, and mitigating burnout syndrome among nursing personnel [13, 14]. Nevertheless, only a very small number of studies have investigated the current status of nurses’ innovation behavior in the context of specialized oncology hospitals, suggesting a moderate level of innovation behavior [15, 16]. Therefore, explaining the influencing factors and promoting the implementation of innovation of nurses in specialized oncology hospitals is necessary.

The triadic reciprocal determinism framework, constituting a foundational theoretical paradigm in social cognitive theory, posits that behavioral patterns emerge through reciprocal interactions among environmental conditions, individual attributes, and behavioral responses [17]. Empirical investigations have delineated multiple predictors of innovation behavior, categorizing determinants into contextual elements (e.g., organizational innovation climate) and individual components (e.g., achievement motivation and self-efficacy) [18–20]. Therefore, it is necessary to explain the mechanisms of nurses’ innovation behavior, in order to provide a theoretical basis for creating evidence-based management protocols and optimizing innovation cultivation strategies for oncology nurses.

## Literature review and research framework

### Organizational innovative climate and nurses’ innovation behavior

Organizational innovative climate constitutes a shared psychological schema among employees regarding institutional support mechanisms for novel initiatives, reflecting collective perceptions of innovation-oriented policies and resource allocation patterns [21]. Within nursing practice contexts, it was defined as nurses’ consensus-based evaluation of the level of support for innovation from the healthcare institution [22]. Empirical investigations have consistently identified organizational innovation climate as a critical contextual determinant influencing innovation behavior development, with cross-cultural validation from diverse healthcare systems [23, 24]. A multicenter study across four Turkish hospitals (*N* = 618 nurses) demonstrated significant correlations between workplace innovation support systems and enhanced innovation behavior frequency and outputs [25]. Parallel findings emerged from a nationwide Chinese cohort (*N* = 1058 frontline nurses), where positive organizational innovation climate is instrumental in fostering nurses’ motivation and awareness for innovation, thus, facilitate the generation of more innovative behavior [26]. Employees would demonstrate heightened innovative propensity when they perceived leadership endorsement for innovation, dedicated temporal and financial resources for innovation, and transparent cross-functional communication infrastructures facilitating idea exchange [27].

### The mediating role of achievement motivation

Achievement motivation conceptualizes the self-regulatory drive that propels individuals toward excellence in competitive scenarios, characterized by persistent pursuit of meaningful objectives and performance optimization [28]. It involves purposeful efforts to complete tasks that are important professionally, motivating them to perform excellently and achieve good outcomes in their activities [29]. The environment factors can directly influence individuals’ achievement motivation [30]. Environmental determinants significantly modulate achievement motivation intensity, as evidenced by Dan et al.‘s investigation (*N* = 515 nurses), where professional practice environment was positively related to achievement motivation [31]. Empirical consensus confirms the positive associations between achievement motivation and innovation behavior [32, 33]. In the context of nursing education, a study involving 499 nursing students found that achievement motivation mediates the relationship between the clinical practice environment and innovative behavior [19]. On this basis, this study proposes the hypothesis that achievement motivation plays a mediating role in organizational innovation climate and nurses’ innovation behavior.

### The mediating role of creative self-efficacy

Self-efficacy, as initially defined by Bandura in 1977, refers to a person’s belief or confidence in their ability to perform specific actions and achieve desired results in particular situations [34]. This core psychological construct underscores the agentic role of personal believe in shaping behavioral selection, effort expenditure, and task persistence [17]. Building upon this foundation, Tierney and Farmer [35] operationalized creative self-efficacy as domain-specific confidence in one’s capacity to generate innovative ideas or produce creative outcomes within innovation-driven environments. Accumulating empirical evidence affirms the positive predictive effect of creative self-efficacy on the manifestation of innovative behavior [36, 37]. Notably, individuals demonstrating elevated creative self-efficacy tend to participate more in innovative activities. Recent studies highlight the mediating role of creative self-efficacy in the influence of organizational factors (e.g., leaderships, supervisor support, clinical practice environment) on innovation behavior among nurses and students [19, 38, 39]. Based on the above considerations, this study proposes the hypothesis that creative self-efficacy plays a mediating role between the organizational innovation climate and nurses’ innovation behavior.

### The chain mediation role of achievement motivation and creative self-efficacy

Achievement motivation and creative self-efficacy constitute critical intrapersonal determinants that synergistically potentiate innovation behavior development. Emerging scholarship elucidates their interconnected dynamics - a cross-sectional analysis of 1,076 undergraduates revealed that self-efficacy is significantly correlated with achievement motivation and can be positively predicted by it [40]. This interdependence aligns with goal regulation theory, where intrinsically motivated individuals exhibit heightened metacognitive awareness and task-specific confidence during complex problem-solving endeavors [41]. Therefore, these results lead us to hypothesize that achievement motivation and creative self-efficacy are two possible mediators that explain the relationship between organizational innovation climate and nurses’ innovation behavior in a sequential manner.

### Research framework

The triadic reciprocal determinism framework further explicates this mechanism through dynamic tripartite interplay: environmental enablers (resource availability, innovation-conducive climates, transformational leadership) reciprocally interact with intrapersonal drivers (motivation, attitudes, believes) to promote the occurrence of behavior [17, 34]. Grounded in this theoretical scaffolding, this study operationalizes organizational innovation climate as the environmental dimension, while positioning achievement motivation and creative self-efficacy as internal factors to examine their effects on innovation behavior.

**Fig. 1:**
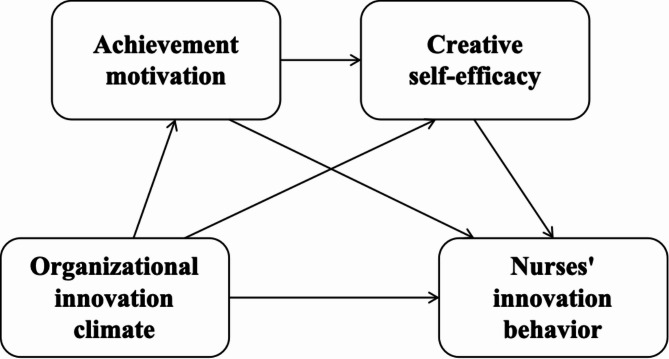
Theoretical framework of this study

Integrating these theoretical and empirical insights, we propose four hypotheses: H1: Organizational innovation climate is positively correlated to innovation behavior among nurses in specialized oncology hospitals. H2: Achievement motivation mediates the relationship between organization innovation climate and nurses’ innovation behavior. H3: Creative self-efficacy mediates the relationship between organization innovation climate and nurses’ innovation behavior. H4: Achievement motivation and creative self-efficacy sequentially mediate the relation between organizational innovation climate and nurses’ innovation behavior. The hypothetical model is shown in Fig. 1.

## Methods

### Design, setting, and participants

An online cross-sectional survey was conducted among nurses from a tertiary specialized oncology hospital in Guangdong province, China from June to September. Utilizing a convenience sampling approach. The inclusion criteria: (1) holding a certificate in nursing qualifications; (2) with minimum 12 months clinical experience in oncology care; (3) voluntary informed consent with full survey completion. The exclusion criteria: (1) internship or probationary nurses; (2) nurses unavailable for duty during data collection phases (including extended leaves, external training assignments, or health-related absences, etc.).

Sample size determination followed Kline’s N: q ratio principle, which prescribes 10–20 observations per estimated parameter to ensure statistical adequacy in structural equation modeling [42]. Given our hypothesized model containing 22 free parameters and anticipating a 10% data attrition rate, the necessary sample size was calculated to range from 242 to 484.

### Measurements

#### General information questionnaire

The research group created a questionnaire to investigate the sociodemographic and professional characteristics of nurses, including gender, age, marital status, working years, highest education level, technical title, whether is a specialist nurse, whether have ever attended an innovation training courses, and whether have ever obtained a patent.

### Nursing organizational innovation climate scale

The Nursing Organizational Innovation Climate Scale, originally validated by Qian et al. [22], was utilized to evaluate nurses’ perception of organizational innovation climate. This instrument comprises three domains containing 21 measurement items: organizational innovation incentive, resource supply, and management and practices. An example item for the scale is, “The leader encourages us to express different opinions and views.” Psychometric evaluations revealed exceptional internal reliability (α = 0.940) and content validity index (0.938). A five-point Likert-type scale is used, with responses from 1 (strongly disagree) to 5 (strongly agree). The total score spans from 21 to 105, with higher scores signifying a stronger perceived climate of organizational innovation. In the current investigation, the reliability coefficient reached 0.960.

### Achievement motivation scale

The Achievement Motivation Scale originated from the work of Nygård and Gjesme [43], with subsequent cross-cultural adaptation and validation for Chinese populations conducted by Ye and Hagtvet [29]. This bidimensional psychometric instrument evaluates two distinct motivational orientations: motive to approach success (MS) and motive to avoid failure (MF). An example item for the MS subscale is, “I feel happy when I finish difficult tasks,” whereas an example item for the MF subscale is, “I am afraid of failure in completing what I consider to be a difficult task.” Psychometric analysis showed reliability coefficients of α = 0.85 for MS and α = 0.87 for MF. Employing a Likert-type response format where 1 signifies “completely inconsistent” and 4 represents “completely consistent”. The composite achievement motivation score is calculated through subtractive scoring (MS − MF), producing a potential spectrum from − 45 to + 45 points, where positive values denote heightened achievement orientation. The present investigation demonstrated robust measurement consistency with an aggregate α coefficient of 0.873.

### Creative self-efficacy scale

The Creative Self-efficacy Scale was developed based on the foundational research by Carmeli and Schaubroeck [44], underwent rigorous cross-cultural adaptation for Chinese populations by Gu et al. This unidimensional instrument employs 8 psychometric items utilizing a Likert-type response format where 1 signifies" strongly disagree” and 5 represents “strongly agree”. Summative scoring yields 8–40 range, with elevated totals denoting heightened creative self-efficacy. Subsequent application by Ju et al. [45], and achieved enhanced reliability in nursing populations (α = 0.932). An example item for the scale is, “Facing difficult tasks, I am absolutely certain that I can complete them in creative ways.” Psychometric evaluation in the current sample revealed satisfied internal consistency (α = 0.952).

### Nurses’ innovation behavior scale

The Nurses’ Innovation Behavior Scale, created by Bao et al. [4]. It encompasses three operational domains totaling 10 measurement items: ideas generation, support obtaining and ideas realization. An example item for the scale is, “You will develop a concrete implementation plan for the new method.” Psychometric robustness was demonstrated through exceptional reliability (α = 0.897) and content validity indices (CVI = 0.910). Utilizing a five-point Likert-type response paradigm (1 = never to 5 = frequently). Total scores (10–50) directly correlate with innovation behavior frequency, where elevated aggregates signify heightened innovation engagement. Total scores (10–50) directly correlate with innovation behavior frequency, where elevated aggregates signify heightened innovation engagement. In our specialized oncology nursing cohort, the instrument exhibited enhanced reliability coefficients (α = 0.950).

### Data collection

Following the formal authorization from nursing administration leadership, data collection was conducted through the certified online questionnaire survey platform “Questionnaire Star” (https://www.wjx.cn/). Participants received comprehensive digital disclosure regarding study objectives, anonymity guarantees, voluntary participation rights, and data privacy. Technical safeguards were implemented to ensure data completeness and uniqueness: (1) participants can only submit the survey unless they completed all the questions, and (2) the questionnaire allowed only one submission per IP address. From an initial cohort of 900 nurses completing the digital assessment, 43 records were systematically excluded based on quality control criteria: completion duration < 180 s or discernible response patterns. Finally, the total number of valid questionnaires was 857, yielding an effective response rate of 95.2%.

### Data analysis

The analytical workflow employed SPSS Statistics v26.0 and AMOS v29.0 for comprehensive data processing. First, Q-Q plots verified approximate normality in the four study variables [46]. Descriptive statistics were conducted to characterized both demographic distributions and the four variables under investigation. Independent samples t-tests and one-way ANOVA were used to analyze the relationships between demographic variables and nurses’ innovation behavior. Factors that affect nurses’ innovation behavior were used as control variables in the subsequent to establish the mediation model. Second, Harman’s single-factor principal component analysis was employed to assess the common method bias [47]. Third, Pearson correlation analysis was employed to investigate the associations between the four variables. Then, confirmatory factor analyses (CFA) were conducted to evaluate the measurement model. Next, structural equation model (SEM) was performed according to the hypothesis, using maximum likelihood estimation with robust standard errors. Finally, bias-corrected bootstrapping with 5,000 resampling iterations was performed to assess the sequential mediation effects between achievement motivation and creative self-efficacy.

## Results

### Demographic characteristics and distribution of nurses’ innovation behavior

Among the 857 oncology nurses analyzed, female practitioners constituting 91.4% (*n* = 783) of the sample versus 8.6% (*n* = 74) male representation. Most nurses were less than 40 years old (79.2%), while marital status data indicated 63.9% (*n* = 548) were married. More than two-thirds of them possessed clinical experience exceeding five years and 795 nurses (92.8%) had attained a baccalaureate degree or above. In terms of technical title, primary nurses accounted for 46.4%. Specialized practice certification prevalence remained limited to 6.0% (*n* = 51) of participants. The detailed general demographic characteristics are presented in Table 1.

Independent sample t test or one-way analysis of variance was used to compare the influence of general demographic variables on nurses’ innovative behavior. There were significant differences in innovation behavior of nurses with different ages, marital status, highest education levels, and technical titles (*P* < 0.05). These results are summarized in Table 1.

**Table 1:** Demographic characteristics and distribution of nurses’ innovation behavior (*n* = 857)

Variables	Frequency (f)	Percentage (%)	t/F	*P*-value
**Gender**				
Male	74	8.6	0.090	0.764
Female	783	91.4		
**Age**				
≤ 30	285	33.3	2.621	0.05
31–40	393	45.9		
41–50	141	16.5		
≥ 51	38	4.4		
**Marital status**				
Single	294	34.3	3.460	0.016
Married	548	63.9		
Divorced	13	1.5		
Widowed	2	0.2		
**Working years**				
1–5	273	31.9	1.623	0.166
6–10	149	17.4		
11–15	207	24.2		
16–20	63	7.4		
≥21	165	19.3		
**Highest education level**				
Advanced diploma or below	62	7.2	15.128	< 0.001
Baccalaureate degree	730	85.2		
Master degree or above	65	7.6		
**Technical title**				
Primary nurse	398	46.4	25.042	< 0.001
Junior nurse	425	49.6		
Senior nurse	34	4.0		
**Whether is a specialist nurse**				
Yes	51	6.0	1.300	0.255
No	806	94.0		

### Common method bias and confirmatory factor analysis

Harman’s single-factor test revealed 9 eigenvalues greater than 1, with the unrotated first factor explaining 26.47% of the total variance, which is below the critical threshold of 40%, indicating acceptable commonality [47].

Confirmatory factor analyses were then conducted to evaluate the four-factor measurement model consisting of organizational innovation climate (latent variable), achievement motivation (observed variable), creative self-efficacy (observed variable), and nurses’ innovation behavior (latent variable). The factor loadings of latent variables were statistically significant and ranged from 0.737 to 0.926. The measurement model also exhibited an acceptable fit with the data [48]: χ²/df = 3.544, GFI = 0.987, RFI = 0.975, NFI = 0.988, CFI = 0.992, TLI = 0.982, and RMSEA = 0.055. The convergent and discriminant validity tests also showed a good fit (see Supplementary Material) [48]. Therefore, a reliable measurement model was, therefore, obtained.

### Descriptive statistics and correlation coefficient

As shown in Table 2, organizational innovation climate demonstrated positive associations across all measured variables: achievement motivation (*r* = 0.212, *P* < 0.01), creative self-efficacy (*r* = 0.472, *P* < 0.01), and nurses’ innovation behavior (*r* = 0.335, *P* < 0.01). Additionally, achievement motivation exhibited small yet significant predictive linkages with both creative self-efficacy (*r* = 0.243, *P* < 0.01) and nurses’ innovation behavior (*r* = 0.170, *P* < 0.01). Notably, creative self-efficacy showed the strongest correlation with nurses’ innovation behavior (*r* = 0.477, *P* < 0.01).

**Table 2:** Means, standard deviations, and variable correlations (*n* = 857)

Variables	M$\:\pm\:$SD	1	2	3	4
1. Organizational innovation climate	3.97$\:\pm\:$0.58	1			
2. Achievement Motivation	0.15$\:\pm\:$0.22	0.212^**^	1		
3. Creative Self-Efficacy	3.45$\:\pm\:$0.68	0.472^**^	0.243^**^	1	
4. Nurses’ innovation behavior	2.81$\:\pm\:$0.75	0.335^**^	0.170^**^	0.477^**^	1

Note: M = Mean; SD = standard deviation; ^**^means *P* < 0.01

### The structure equation model

**Fig. 2:**
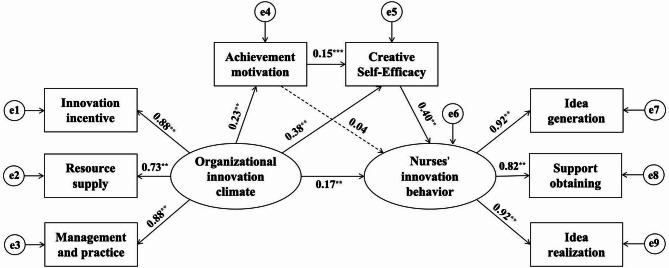
Structure equation model of nurses’ innovation behavior (*n* = 857). Note: ^**^means *p* < 0.01; Dashed lines indicate that the indirect effect was not significant; Control variables predicting nurse innovation behavior (i.e., age, highest education level, marital status and technical title) are not presented for simplicity reasons

The hypothesized theoretical framework was operationalized through structural equation modeling, demonstrating excellent global fit indices: χ²/df = 3.459, GFI = 0.971, RFI = 0.955, NFI = 0.970, CFI = 0.978, TLI = 0.967, and RMSEA = 0.054, collectively satisfying Byrne’s [48] stringent psychometric thresholds. As illustrated in Fig. 2, organizational innovation climate exerted a notably positive impact on nurses’ innovation behavior (*b* = 0.17, *p* < 0.01), thereby confirming H1. Organizational innovation climate also had a positive impact on both achievement motivation (*b* = 0.23, *p* < 0.01) and creative self-efficacy (*b* = 0.376, *p* < 0.01). While achievement motivation significantly predicted creative self-efficacy (*b* = 0.168, *p* < 0.01), its direct effect on nurses’ innovation behavior proved nonsignificant (*b* = 0.04, *p* > 0.05). Notably, creative self-efficacy manifested the strongest direct effect on nurses’ innovation behavior (*b* = 0.40, *p* < 0.01).

As shown in Table 3, the total effect of organizational innovation climate on nurses’ innovation behavior was statistically significant (β = 0.262, 95% 95% CI [0.219, 0.308]), with indirect effects contributed to 53.01% of the total variance explained. Among the indirect pathways, creative self-efficacy emerged as the dominant mediator, explaining 43.51% of the total effect (β = 0.114, 95% CI [0.091, 0.141]), thereby confirming H3. In contrast, the single mediation through achievement motivation was nonsignificant (β = 0.008, 95% CI [-0.002, 0.020]), leading to the rejection of H2. Notably, the sequential mediation pathway (organizational innovation climate → achievement motivation → creative self-efficacy → nurses’ innovative behavior) demonstrated a small yet statistically meaningful effect (β = 0.012, 95% CI [0.007, 0.018]), accounting for 4.58% of the total effect and supporting H4.

**Table 3:** Direct and indirect effects (*n* = 857)

Effect	B	SE		Bootstrapping 95% CI	
				Lower	Upper
Total effect of OIC to NIB	0.262^**^	0.027		0.219	0.308
Direct effect of OIC to NIB	0.128^**^	0.027		0.084	0.172
Indirect effect of OIC to NIB	0.134^**^	0.015		0.111	0.160
Indirect effect 1: OIC→AM→NIB	0.008	0.007		-0.002	0.020
Indirect effect 2: OIC→CSE→NIB	0.114^**^	0.015		0.091	0.141
Indirect effect 3: OIC→AM→CSE→NIB	0.012^**^	0.003		0.007	0.018

Note: OIC = organizational innovation climate; AM = achievement motivation; CSE = creative self-efficacy; NIB = nurses’ innovation behavior; CI = confidence interval; ^**^means *p* < 0.01

## Discussion

This study examined the current state of nurses’ innovation behavior within a specialized oncology hospital setting and analyzed how organizational innovation climate influences such behavior through the mediating factors of achievement motivation and creative self-efficacy. The hypotheses of our study received partial support. In our study, the nurses’ innovation behavior scored below moderate levels (2.81 ± 0.75), lower than values reported in studies from Chinese general hospitals [20, 26], Greece [49], and Pakistan [50]. This discrepancy may be attributed to the unique challenges faced by oncology nurses, including managing patients with complex, high-acuity conditions and heavy workloads, which may limit opportunities for innovative practices [7, 8]. The results underscore the need for targeted interventions to enhance innovation behavior in this specialized nursing population. Environmental and individual characteristics are both important factors that influencing nurses’ innovation behavior [18]. Thus, this study explained the possible mechanisms on how organizational innovation climate affects nurses’ innovation behavior through achievement motivation and creative self-efficacy.

First, the analysis revealed a notable positive correlation between nurses’ innovation behavior and organizational innovation climate (β = 0.335, *P* < 0.01), demonstrating that enhanced perceptions of organizational support for innovation corresponded to improved innovative practices among oncology nurses—a finding consistent with prior researches in general healthcare settings [25, 26]. Organizational innovation climate, defined as nurses’ sustained perception of workplace support for creative initiatives [51], functions as a critical environmental determinant shaping longitudinal innovation patterns. A qualitative study identified two opposing forces in this dynamic: restrictive factors (e.g., excessive clinical workloads, resource scarcity) and facilitative elements (e.g., interdisciplinary collaboration, leadership endorsement) [52]. A robust innovation climate systematically fosters nurse-driven creativity through optimized resource allocation, interdisciplinary exchange platforms, innovation-specific funding, and opportunities for career development [53]. These institutional safeguards collectively promote nurses to engage in innovative activities and stimulate the intrinsic enthusiasm and initiative to develop innovative behavior.

Based on Bandura’s triadic reciprocal determinism framework, this investigation operationalized a serial mediation analysis elucidate the psychosocial mechanisms between organizational innovation climate and nurses’ innovation behavior. Our results revealed achievement motivation’s lack of significant mediating influence between organizational innovation climate and nurses’ innovation behavior, despite confirming direct organization innovation climate effects on both achievement motivation and innovation behavior. This finding aligns with the research result by Xiang et al. [19] in nursing students. This could be interpreted by Atkinson’s achievement motivation model, which posits that achievement motivation is composed of the MS and MF, and their effects on generating innovative behavior are opposite [54]. In this study, the near-parity of these opposing forces in our participants (total achievement motivation score: M = 0.15 ± 0.22) likely exerted negligible directional influence on innovation praxis, effectively neutralizing achievement motivation’s mediating capacity.

Furthermore, this analysis verified that creative self-efficacy significantly mediates the relationship between the organizational innovation climate and nurses’ innovative behavior, demonstrating that enhanced perceptions of organizational support amplify nurses’ confidence in their creative capabilities, thereby fostering innovative behaviors, which is identical to prior empirical evidence [55, 56]. Creative self-efficacy has been identified as a crucial process variable in understanding how organizational elements impact creative results [36]. Rooted in Bandura’s self-efficacy theory, these findings emphasize that nurses possessing stronger creative self-efficacy exhibit greater resilience in navigating complex clinical challenges [57]. The Pygmalion effect further elucidates this mechanism [58]: when organizational leaders actively endorse innovation through resource allocation and verbal reinforcement, they cultivate a psychological climate that elevates nurses’ confidence and belief in innovation, which promotes the generation of innovation behavior.

Notably, the chain mediation analysis substantiated a significant sequential pathway through which organizational innovation climate influences nursing innovation behavior—first enhancing achievement motivation, which subsequently amplifies creative self-efficacy, ultimately driving innovative behaviors. This triadic mechanism aligns with Bandura’s social cognitive theory, wherein environmental stimul, cognitive factors, and motivational states dynamically interact to shape behavioral outcomes [37]. While prior research established unidirectional links between self-efficacy and achievement motivation [19, 59], our findings introduced new insights indicating that achievement motivation positively impacts creative self-efficacy in the area of nursing innovation, which extend this paradigm by demonstrating their synergistic mediation. Nurses perceiving organizational support experience heightened goal-directed motivation, which strengthens their perceived capacity to execute innovative solutions. This enables tangible improvements like refining clinical procedures, developing new tools, or creating communication strategies to address workflow inefficiencies and patient care challenges—directly enhancing the quality of care and patient outcomes [60, 61]. Therefore, the identification of this chained pathway underscores the necessity of multistage interventions targeting both motivational activation and self-efficacy cultivation in specialized oncology settings.

### Practical implications

This research results have certain theoretical and practical significance. In terms to the theoretical significance, this study elucidated that organizational climate can influence nurses’ innovative behavior through achievement motivation and creative self-efficacy, which expands the application field of the triadic reciprocal determinism framework and deeply explores the internal mechanism of individual factors, offering a theoretical basis for nursing administrators to implement targeted interventions that enhance innovation practices among oncology nurses.

In practical sense, firstly, it is necessary to cultivate a robust, sustainable ecosystem for clinical creativity. Central to this effort is the establishment of comprehensive innovation infrastructure, including evidence-based training programs in design thinking and translational research methodologies, to address critical competency gaps [62, 63]. Concurrently, fostering interdisciplinary collaboration platforms to create a cooperative working environment, and encouraging nurses to iteratively develop solutions alongside cross-functional partners [64]. Complementing these structural supports, a reward system can be established, for nurses who actively engage in innovation, providing them with financial incentives and preferential career development opportunities [65]. By synergistically addressing resource accessibility, collaborative capacity, and motivational drivers, the initiative of nurses’ innovation could be active. In addition, nursing administrators should implement competency development programs that systematically cultivate achievement-oriented mindsets, such as pairing oncology nurses with translational research mentors to enhance professional mastery and goal attainment [66]. The establishment of specialized nursing innovation posts may also stimulate nurses’ motivation for innovation to promote their career development [67]. To optimize creative self-efficacy, nursing managers can help nurses to divide difficult innovation work into easy-accomplished tasks to increase their confidence and sense of achievement in innovation. Vicarious learning as the key mechanisms in self-efficacy formation should be prioritized for implementation, for instance, inviting nursing innovation experts to do successful experience sharing.

### Limitations and future research direction

This study acknowledges several limitations. First, the non-probabilistic sampling strategy restricted participant recruitment to a single oncology hospital in Guangdong Province, China, potentially constraining the external validity of findings. Therefore, future study warrant implementation across multi-center research with stratified random sampling method. Second, the observational design inherently limits causal inference despite structural equation modeling’s capacity for simultaneous variable analysis. Future research could use longitudinal or experimental data to explore the causal relations between the model variables. Third, the study utilized self-reported measurements, potentially leading to subjective findings. Subsequent research could enhance objectivity through triangulation with objective proxies (e.g., number of patents) and supervisor evaluations of implemented innovations. Finally, this research results validate part of the pathways in the hypothesized model, future investigations could enhance understanding by incorporating qualitative research methods.

## Conclusion

The finding substantiates organizational innovation climate as a critical determinant of innovation behavior among oncology nurses, and through a sequential psychosocial mechanism wherein environmental support first enhances achievement motivation, which subsequently amplifies creative self-efficacy, ultimately driving innovative behavior. These findings support Bandura’s triadic reciprocal determinism, demonstrating how environmental inputs, cognitive factors, and motivational states interact dynamically to shape behavioral behavior in oncology nursing contexts. Effective strategies such as establishing institutional support systems, fostering motivational resilience and creative self-efficacy should be implemented to promote nurses’ innovation behavior, ultimately enhancing quality of care through nurse-driven process improvements and therapeutic innovations.

## Electronic supplementary material

Below is the link to the electronic supplementary material.

Supplementary Material 1

## Data Availability

The data that support the findings of this study are available on request from corresponding author Prof. Huiying Qin upon reasonable request.
