# Supplementary material for: Organizational innovation climate and nurses’ innovation behavior in a specialized oncology hospital: the chain mediation of achievement motivation and creative self-efficacy
Source: BMC Nurs. 2025 Jul 21;24:951. doi: 10.1186/s12912-025-03595-8 (PMC12278570; doi:10.1186/s12912-025-03595-8)
Supplement: Supplementary file 1 — Supplementary Material 1 [file 12912_2025_3595_MOESM1_ESM.docx]

Table S1 The CR, AVE, MSV and correlation of variables

| **Variables** | CR | AVE | MSV | 1 | 2 | 3 | 4 |
| --- | --- | --- | --- | --- | --- | --- | --- |
| 1. OIC | 0.873 | 0.697 | 0.219 | 0.835 |  |  |  |
| 2. AM | - | - | 0.064 | 0.228** | - |  |  |
| 3. CSE | - | - | 0.219 | 0.468** | 0.253** | - |  |
| 4. NIB | 0.919 | 0.790 | 0.212 | 0.374** | 0.182** | 0.46** | 0.889 |

Note: OIC = organizational innovation climate, is a latent variable; AM = achievement motivation, is an observed variable; CSE = creative self-efficacy, is an observed variable; NIB = nurses’ innovation behavior, is a latent variable; CR= composite reliability; AVE= average variance extracted; MSV=maximum shared variance; diagonal is the square root of AVE. ** means *P*<0.01.
